# Comparison of BISAP, Ranson, MCTSI, and APACHE II in Predicting Severity and Prognoses of Hyperlipidemic Acute Pancreatitis in Chinese Patients

**DOI:** 10.1155/2016/1834256

**Published:** 2016-11-02

**Authors:** Lixin Yang, Jing Liu, Yun Xing, Lichuan Du, Jing Chen, Xin Liu, Jianyu Hao

**Affiliations:** ^1^Department of Gastroenterology, Beijing Chaoyang Hospital, Capital Medical University, Beijing 100020, China; ^2^Beijing First Hospital of Integrated Chinese and Western Medicine, Beijing, China; ^3^Hebei Xianghe People's Hospital, Xianghe, China

## Abstract

In recent years, with the developing of living standard, hyperlipidemia becomes the second major reason of acute pancreatitis. It is important to predict the severity and prognosis at early stage of hyperlipidemic acute pancreatitis (HLAP). We compared the BISAP, Ranson, MCTSI, and APACHE II scoring system in predicting MSAP and SAP, local complications, and mortality of HLAP. A total of 326 diagnosed hyperlipidemic acute pancreatitis patients from August 2006 to July 2015 were studied retrospectively. Our result showed that all four scoring systems can be used to predict the severity, local complications, and mortality of HLAP. Ranson did not have significant advantage in predicting severity and prognosis of HLAP compared to other three scoring systems. APACHE II was the best in predicting severity of HLAP, but it had shortcoming in predicting local complications. MCTSI had outstanding performance in predicting local complications, but it was poor in predicting severity and mortality. BISAP score had high accuracy in assessment of severity, local complications, and mortality of HLAP, but the accuracy still needs to be improved in the future.

## 1. Introduction

Recently, with the change of people's living condition and life style, hyperlipidemia comes to the second major reason of acute pancreatitis. A study involving 2416 cases diagnosed with acute pancreatitis (AP) from 2006 to 2010 in Beijing found that 255 (10.36%) cases were hyperlipidemic acute pancreatitis (HLAP) [1]. An analysis carried by Xu et al. claims that, in the period of 2012 to 2014, HLAP accounted for 19.1% of total AP [2]. Compared with NHLAP, HLAP are characterized by critical condition and high recurrence rate [3]. So, it is important to predict the severity and prognosis of HLAP at early stage, which is beneficial for individualized treatment and prognosis.

There are four frequently used scoring systems of AP, including BISAP (bedside index for severity in acute pancreatitis), Ranson score, MCTSI (modified CT severity index), and APACHE II (acute physiology and chronic health evaluation scoring system). To our knowledge, there is no large-population-based study in assessment of severity and prognosis of HLAP. In this paper, a total of 326 cases diagnosed with HLAP from 2007 to 2015 admitted to a single center were retrospectively analyzed to compare the prediction value of four scoring systems.

## 2. Material and Method

### 2.1. Data

We retrospectively analyzed a series of 326 patients diagnosed with HALP who were admitted to Beijing Chao-Yang Hospital, Capital Medical University, in a period of August 2007 to July 2015 (184 males, 142 females; age ranging from 14 to 85; mean age of 44 years). Of the 326 patients included, 65 (19.9%) had moderately severe acute pancreatitis (MSAP), 27 (8.3%) had severe acute pancreatitis (SAP), 28 (8.59%) had local complications, and 9 died (2.8%).

Local complications include acute peripancreatic fluid collection, pancreatic pseudocyst, walled-off necrosis, infected necrosis, pleural effusion, intestinal fistula, and pancreatic pseudocyst hemorrhage. Of the 28 patients with local complications, 21 had two or more local complications. 72 patients had a relapse, and the recurrence rate is 22.09%. Among them 15 (7.67%) had 3 or more recurrent relapses.

### 2.2. Diagnostic Criteria and Scoring System

The diagnostic criteria of acute pancreatitis referred to Chinese guideline for diagnosis and treatment of acute pancreatitis and Atlanta Classification of Acute Pancreatitis [4, 5].

The diagnosis of acute pancreatitis, whether in the presence or absence of underlying chronic pancreatitis, requires two of the following three features: (1) abdominal pain suggestive strongly of acute pancreatitis, (2) serum amylase and/or lipase activity at least 3 times greater than the upper limit of normal, and (3) characteristic findings of acute pancreatitis on transabdominal ultrasonography, contrast-enhanced ECT, or magnetic resonance imaging (MRI).

The grading of severity of AP referred to Chinese guideline for diagnosis and treatment of acute pancreatitis as follows:

1. MAP meets AP diagnostic criteria. MAP requires one of the following three features: no organ failure; no local or systemic complications; and Ranson score < 3 points; APACHE II score < 8; BISAP score < 3 points; and MCTSI score < 4 points.
2. MSAP meets AP diagnostic criteria. At the same time, MSAP should meet one of the following conditions: (1) Ranson score ≥ 3 points; (2) APACHE score ≥ 8 points; (3) BISAP score ≥ 3 points; (4) MCTSI ≥ 4 points; (5) transient organ failure (<48 h); (6) pseudocyst, pancreatic fistula, or pancreatic abscess that needs surgical operation.
3. SAP meets AP diagnostic criteria and have the presence of persistent (>48 h) organ failure (single or multiple organ) and the modified Marshall score ≥ 2 points.

HLAP was diagnosed if serum triglycerides (TG) reached 11.3 mmol/L, or TG was more than 5.56 to 11.3 mmol/L accompanied by chylemia, excluding other etiologies of AP (gallstone, drug, infection, etc.) [6, 7].

BISAP and APACHE II score were calculated in 24 hours after admission. Ranson score was calculated in 48 hours. MCTSI score was calculated in patients with contrast-enhanced CT scan within 3 days of onset. The score is calculated based on the worst value of each criteria [8–10].

Local complications were evaluated by contrast-enhanced computed tomography (CECT). CECT scans were retrospectively and independently reviewed by two experienced abdominal radiologists who were unaware of presenting signs and symptoms or of patient outcomes. Kappa statistic was calculated for measuring agreement between two radiologists and the result indicates good agreement. We obtained approval from Institutional Review Board of Beijing Chao-Yang Hospital for this study.

### 2.3. Statistical Analysis

The data was statistically analyzed using SPSS software 19.0. BISAP, Ranson, MCTSI, and APACHE II were compared in predicting severity, location complications, and mortality of HLAP, using chi-square testing, Fisher's exact probability test, and receiver operating characteristic curve. Odds ratio (OR), sensitivity, specificity, positive predictive value (PPV), positive likelihood ratio (PLR), Youden index, and area under ROC curve (AUC) were calculated. A *p* value < 0.05 was considered as statistically significant.

## 3. Result

### 3.1. Predicting Value of Four Scoring Systems in HLAP

Tables 1, 2, and 3 show that the incidence of MSAP and SAP, local complications, and mortality in patients with BISAP score ≥ 3, Ranson score ≥ 3, APACHE II score ≥ 8, and MCTSI score ≥ 4 were significantly higher than those in BISAP score < 3, Ranson score < 3, APACHE II score < 8, and MCTSI < 4 (*p* < 0.05).

### 3.2. Comparison of the Four Scoring Systems in Predicting Severity of HLAP

**Figure 1:**
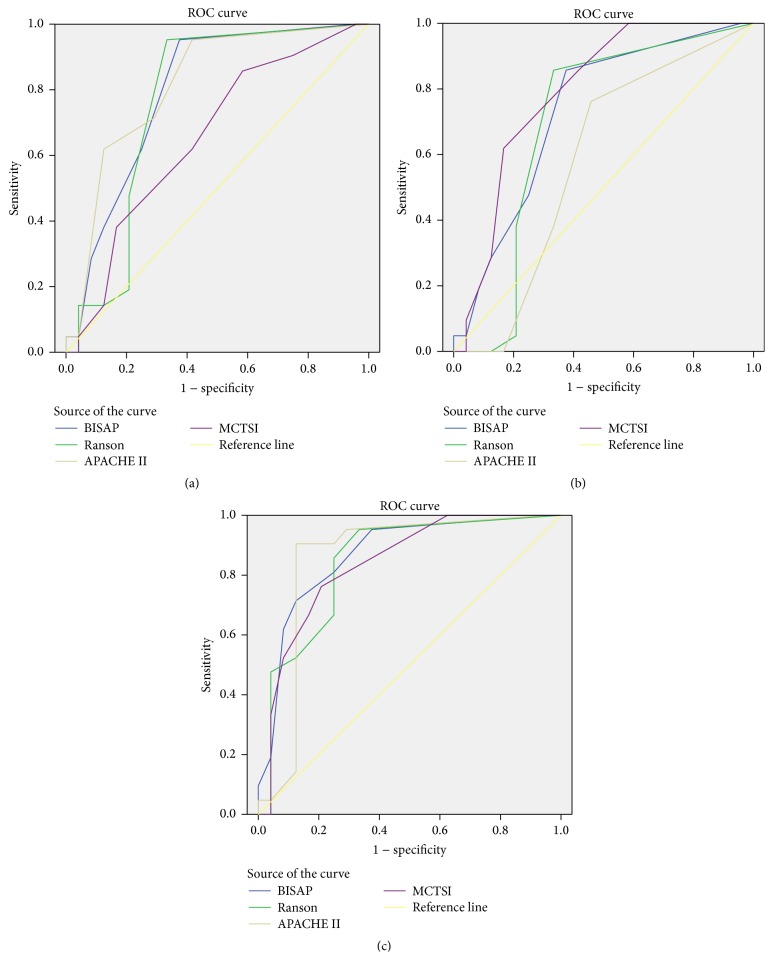
ROC curve of 4 scoring systems in predicting severity, local complications, and mortality of HLAP. (a) ROC curve of 4 scoring systems in predicting severity of HLAP. (b) ROC curve of 4 scoring systems in predicting local complications of HLAP. (c) ROC curve of 4 scoring systems in predicting mortality of HLAP.

Table 4 and Figure 1(a) show the sensitivity, specificity, PPV, PLR, Youden index, and AUC of BISAP, Ranson, MCTSI, and APACHE II in predicting severity of HLAP. In assessment of severity, the sensitivity and AUC of APACHE II were 57% and 0.814, which were the highest. BISAP was second with sensitivity of 54%. And the AUC of BISAP was 0.795.

### 3.3. Comparison of the Four Scoring Systems in Predicting Local Complications of HLAP

Table 4 and Figure 1(b) show the sensitivity, specificity, PPV, PLR, Youden index, and AUC of BISAP, Ranson, MCTSI, and APACHE II in predicting location complications of HLAP. In assessment of local complications, the sensitivity and AUC of MCTSI were 68% and 0.791, which were the highest. BISAP was second with AUC of 0.731.

### 3.4. Comparison of the Four Scoring Systems in Predicting Mortality of HLAP

Table 4 and Figure 1(c) show the sensitivity, specificity, PPV, PLR, Youden index, and AUC of BISAP, Ranson, MCTSI, and APACHE II in assessment of mortality of HLAP. In assessment of mortality, the sensitivity and AUC of BISAP were 89% and 0.867, which were the highest. The second was APACHE II with AUC of 0.854. Both APACHE II and BISAP had the highest sensitivity of 89%.

## 4. Discussion

Compared with NHLAP, HLAP has the following features: (1) high recurrence rate: the higher the blood lipid level is, the greater the possibility of recurrence; serum TG lower than 5.56 mmol/L can prevent episodes of pancreatitis; (2) serum TG above 11.3 mmol/L; (3) xanthomata in the limbs, buttocks and back, retinal lipemia, hepatosplenomegaly, and fatty liver which can be found in patients with severe hypertriglyceridemia (HTG) because of lipid deposition; (4) patients with HLAP having younger age of onset. Uncontrolled diabetes, obesity, alcoholism, pregnancy, family history of hyperlipidemia are thought to be the risk factors for HLAP [11, 12].

It has been reported that the incidence of sever acute pancreatitis (SAP) and organ dysfunction, recurrence rate, and mortality of HLAP were significantly higher than those of acute biliary pancreatitis [3]. So, it is extremely important to predict and evaluate the severity of HLAP at early stage. The clinical scoring system is a practical tool for the doctor to find potential patients who need intensive care. However, there is little research on this aspect. A total of 129 cases of HLAP show that it is fairly accurate to predict SAP, complications, organ failure, and prognostic of HLAP, using BISAP, Ranson, SIRS, and MCTSI (area under the curve ranges from 0.938 to 0.668) [13, 14].

BISAP, Ranson, APACHE II, and MCTSI are the most commonly used scoring systems to evaluate acute pancreatitis, but they still have limitations. Firstly, APACHE II scoring system is not convenient to operate, because it has too many parameters to collect. Secondly, different scoring systems contain common parameters; for example, SIRS is a composite parameter used in both BISAP and APACHE II. However, different scoring systems have different way to evaluate one parameter. Taking blood urea nitrogen (BUN) as an example, Ranson takes the increasing level of BUN as criteria, but BISAP takes the absolute value as the criteria. So it takes a lot of time using variety of scoring systems to predict the prognosis.

We hope to select one scoring system that can predict the prognosis of HLAP accurately and easily. So we compared the accuracy of four scoring systems in predicting severity, complications, and mortality.

Our study showed that the incidence of MSAP and SAP, local complications, and mortality were significantly higher in BISAP score ≥ 3, Ranson score ≥ 3, APACHE II score ≥ 8, and MCTSI score ≥ 4 than in BISAP score < 3, Ranson score < 3, APACHE II score < 8, and MCTSI < 4 (*p* < 0.05). Therefore, all four scoring systems can be used to predict the severity, local complications, and mortality of HLAP.

In 1974, Ranson et al. selected 11 indicators associated with the severity of AP by screening 43 clinical and biochemical indicators [8]. Our study showed that Ranson score was poor in predicting severity and prognosis of HLAP. The AUC of it ranked the third in every aspect we considered. In assessment of MSAP and SAP and mortality, the AUC of Ranson was higher than MCTSI. And Ranson had the lowest positive predictive value, positive likelihood ratio, and Youden index compared with other scoring systems. In assessment of local complications, Ranson was only better than APACHE II.

APACHE II is a frequently used scoring system to assess severity of AP. It consists of three parts, namely, acute physiology score, age, and chronic health score. Our study showed that the APACHE II had highest accuracy in predicting MSAP and SAP and did a good job in predicting mortality. But APACHE II was poor in assessment of local complications. In assessment of MSAP and SAP, the AUC and Youden index of APACHE II were 0.814 and 0.46, respectively, which were the highest among these four scoring systems. In assessment of mortality, it had the second highest AUC (0.854) and Youden index (0.67), lower than BISAP. In terms of assessing local complications, APACHE II had the lowest AUC (0.580), PPV (15), PLR (2.0), and Youden index (0.21).

MCTSI is a clinical radiological imaging scoring system for evaluating the mortality and local complications of AP. Contrast-enhanced CT is the gold standard for diagnosis of necrotizing pancreatitis and acute peripancreatic fluid collection [10]. In our study, MCTSI had outstanding performance in predicting location complications, with AUC of 0.791. However, it was poor in predicting severity and mortality. The sensitivity and AUC of MCTSI in assessment of MSAP and SAP and mortality were 36%, 0.654 and 78%, 0.839, respectively, which were lower than the other three scoring systems. According to the retrospective study by Bollen et al., there were no significant differences in prediction accuracies between 7 CT scoring systems and two clinical scoring systems (APACHE II, BISAP), so contrast-enhanced CT scan was not recommended for severity assessment on admission [15]. That conforms to our study.

BISAP was proposed to construct a simple and accurate clinical scoring system to estimate the mortality risk of AP at early stage. BISAP analyzed the in-hospital mortality risk of AP using classification and regression tree analysis, and finally five variables were selected. This scoring system was established from a study involving 17992 cases diagnosed with AP from 212 hospitals. Compared with the APACHE II, the validity of the BISAP score was confirmed using a data of 18256 cases diagnosed with AP from 177 hospitals [9]. Singh et al. applied BISAP to evaluate the severity and mortality in a prospective study enrolling 397 cases diagnosed with AP and came to the same conclusion that BISAP was effective to evaluate the severity of AP [16]. A study by Papachristou et al. indicated that the sensitivity and specificity of BISAP were not worse than “traditional” scoring system (Ranson score, APACHE II, and MCTSI) [17]. In our study, BISAP had high accuracy in predicting MSAP and SAP, local complications, and mortality. In predicting mortality of HLAP, the sensitivity (89%), Youden index (0.69), and AUC (0.867) were the highest in all scoring systems. In predicting MSAP and SAP, the AUC of BISAP was slightly lower than APACHE II. In predicting local complications, the AUC of BISAP was slightly lower than MCTSI. So, BISAP performed better than other three scoring systems in every aspect we compared in this study.

In conclusion, Ranson did not have significant advantage in predicting severity and prognosis of HLAP. APACHE II was the best in predicting severity, but it had shortcoming in predicting local complications. MCTSI had outstanding performance in predicting local complications. But it is poor in predicting severity and mortality. BISAP score had high accuracy in predicting severity, local complications, and mortality of HLAP. And BISAP score has the advantages of having less parameters and being easy to operate. Further studies in comparison to these four scoring systems appear to be needed, since our study was a single-center study. Nevertheless, we recommended using BISAP to predict the severity and prognosis of HLAP.

However, the accuracy of BISAP should be promoted in further study. In assessment of severity, for example, the AUC value of APACHE II was the highest (0.814), while BISAP was slightly lower (0.795). Mounzer et al. had shown that the current scoring system for acute pancreatitis had reached the maximum efficacy in prediction of organ failure. It was more accurate to predict the severity of combined use of several scoring systems, but it was inconvenient for clinical practice. Unless new scoring system was proposed, it was difficult to improve the prediction accuracy of AP [18].

Another study showed that, compared with NHLAP, the C-reaction protein (CRP) in patients with HLAP was significantly higher on days 1, 2, 3, 4, and 6. That means CRP has predictive value in patients with HLAP [13, 14]. We believed that the diagnostic accuracy will be improved by combining the scoring systems with biochemical parameters correlating with the severity of HLAP, such as CRP and high density lipoprotein. And that is the direction of our further studies [13, 19].

## Figures and Tables

**Table 1:** Analysis of the four scoring systems in predicting severity of HLAP.

Scoring system	MSAP and SAP	MAP	Total	OR	*χ* ^2^	*p*
BISAP						
≥3	48	23	71	10.0	73.1	<0.05
<3	44	211	255			
Ranson						
≥3	43	37	80	4.7	34.1	<0.05
<3	49	197	246			
APACHE II						
≥8	52	26	78	10.4	74.8	<0.05
<8	40	208	248			
MCTSI						
≥4	33	17	50	8.2	45.6	<0.05
<4	59	217	276			

MAP = mild acute pancreatitis						

**Table 2:** Analysis of the four scoring systems in predicting location complications of HLAP.

Scoring system	Local complication	No local complication	Total	OR	*χ* ^2^	*p*
BISAP						
≥3	15	56	71	5.0	18.2	<0.05
<3	13	242	255			
Ranson						
≥3	16	64	80	4.9	17.6	<0.05
<3	12	234	246			
APACHE II						
≥8	12	66	78	2.6	6.0	<0.05
<8	16	232	248			
MCTSI						
≥4	19	31	50	18.2	65.1	<0.05
<4	9	267	276			

**Table 3:** Analysis of the four scoring systems in predicting mortality of HLAP.

Scoring system	Mortality	Survival	Total	OR	*χ* ^2^	*p*
BISAP						
≥3	8	63	71	32.3	24.5	<0.05
<3	1	254	255			
Ranson						
≥3	7	73	80	11.7	14.2	<0.05
<3	2	242	246			
APACHE II						
≥8	8	70	78	28.2	21.5	<0.05
<8	1	247	248			
MCTSI						
≥4	7	43	50	21.7	27.1	<0.05
<4	2	275	276			

**Table 4:** Comparison of 4 scoring systems in predicting severity, local complications, and mortality of HLAP.

Scoring system	Sensitivity	Specificity	PPV (%)	PLR	Youden index	AUC	95% CI
*MSAP and SAP*							
BISAP	54	86	68	3.9	0.4	0.795	0.660–0.929
Ranson	46	84	54	2.9	0.3	0.766	0.616–0.916
APACHE II	57	89	67	5.2	0.46	0.814	0.686–0.942
MCTSI	36	94	66	6	0.3	0.654	0.492–0.815
*Location complications*							
BISAP	54	81	21	2.8	0.35	0.731	0.581–0.881
Ranson	57	79	20	2.7	0.36	0.698	0.532–0.865
APACHE II	43	78	15	2.0	0.21	0.580	0.407–0.753
MCTSI	68	90	38	6.8	0.58	0.791	0.656–0.925
*Mortality*							
BISAP	89	80	15	4. 5	0.69	0.867	0.758–0.976
Ranson	78	77	9	3.4	0.55	0.842	0.722–0.962
APACHE II	89	78	10	4.0	0.67	0.854	0.724–0.984
MCTSI	78	86	14	5.6	0.64	0.839	0.721–0.957
